# Baseline Characteristics of Participants in the Exercise for Cancer to Enhance Living Well (EXCEL) Study: A Canada‐Wide Rural–Urban Analysis

**DOI:** 10.1002/cam4.71629

**Published:** 2026-03-20

**Authors:** Jonathan L. Low, Julianna Dreger, Chad W. Wagoner, Emma McLaughlin, Margaret L. McNeely, Melanie R. Keats, Daniel Santa Mina, Linda Trinh, Kristin L. Campbell, Isabelle Doré, Heejae Lee, Colleen A. Cuthbert, Lauren C. Capozzi, Daniel Sibley, Thomas B. Christensen, Alexia Piché, Kelly Mackenzie, Carol Yin, S. Nicole Culos‐Reed

**Affiliations:** ^1^ Faculty of Kinesiology University of Calgary Calgary Alberta Canada; ^2^ Riddell Center for Cancer Immunotherapy, Cumming School of Medicine University of Calgary Calgary Alberta Canada; ^3^ Department of Kinesiology, Recreation, and Sport Studies University of Tennessee ‐ Knoxville Knoxville Tennessee USA; ^4^ Department of Physical Therapy University of Alberta Edmonton Alberta Canada; ^5^ Faculty of Health, School of Health and Human Performance Dalhousie University Halifax Nova Scotia Canada; ^6^ Faculty of Kinesiology and Physical Education University of Toronto Toronto Ontario Canada; ^7^ Department of Physical Therapy, Faculty of Medicine University of British Columbia Vancouver Alberta Canada; ^8^ School of Kinesiology and Physical Activity Sciences, Faculty of Medicine Université de Montréal Montréal Quebec Canada; ^9^ School of Public Health Université de Montréal Montréal Quebec Canada; ^10^ Université de Montréal, Hospital Research Centre (CRCHUM) Montréal Quebec Canada; ^11^ Faculty of Nursing University of Calgary Calgary Alberta Canada; ^12^ Rehabilitation Oncology, Physiatry, AE Child Comprehensive Cancer Center, Cancer Care Alberta, Alberta Health Services Calgary Alberta Canada; ^13^ Cancer Rehabilitation, BC Cancer Kelowna British Columbia Canada; ^14^ Department of Oncology, Cumming School of Medicine University of Calgary Calgary Alberta Canada; ^15^ Department of Psychosocial Resources, AE Child Comprehensive Cancer Centre, Cancer Care, Alberta Health Services Calgary Canada

**Keywords:** baseline characteristics, cancer survivorship, exercise oncology, geographic disparities, rural health, urban health

## Abstract

**Introduction:**

Exercise interventions improve quality of life and survival for individuals living with and beyond cancer (ILWBC), yet equitable access remains limited. Evidence on characteristics of who enrolls in exercise oncology programs is scarce, leaving gaps for equity‐focused recruitment and scale‐up, particularly in rural and underserved settings. The EXercise for Cancer to Enhance Living Well (EXCEL) study offers a unique opportunity to examine these issues across a Canada‐wide cohort.

**Methods:**

EXCEL is an 8–12‐week tailored exercise intervention delivered primarily to ILWBC in rural/remote communities, with additional enrollment of urban participants lacking exercise oncology resources. Adults with any cancer type or stage were eligible if pre‐treatment, receiving treatment, or within 3 years post‐treatment. This analysis describes baseline demographic, lifestyle, medical, and fitness factors by rural versus urban residence using descriptive statistics.

**Results:**

Of 1495 participants enrolled in the EXCEL program (rural *n* = 1085; urban *n* = 400), baseline characteristics differed modestly by geography. Age did not differ significantly between rural and urban participants. Rural participants were more often male, had lower educational attainment, and demonstrated higher BMI than urban participants. Urban participants exhibited greater ethnic diversity and higher levels of education. Physical activity levels were similar with 78% classified as physically active at baseline.

**Conclusion:**

This Canada‐wide baseline analysis reveals rural–urban variations in age, treatment status, disease burden, education, and lifestyle, yet comparable physical activity and functional capacity levels. These findings provide descriptive evidence to inform recruitment strategies and considerations for exercise oncology program delivery across geographic settings.

## Abstract 2

Leveraging a uniquely large Canada‐wide cohort (*N* = 1495) of individuals living with and beyond cancer (ILWBC), we stratified participants in the EXCEL trial by rural–urban geography. Our findings demonstrate that rural ILWBC are unique in baseline sociodemographic and medical characteristics compared with urban participants yet have comparable physical readiness for exercise oncology programs.

## 1 Introduction

Exercise interventions enhance quality of life for individuals living with and beyond cancer (ILWBC), improving treatment‐related adverse effects, cancer‐related fatigue, mental health, and physical function [1, 2]. Exercise has also been associated with survival [3, 4], with a recent randomized trial demonstrating improved survival among ILWBC assigned to exercise versus health education [5]. Despite these benefits, access to exercise oncology programs remains limited, particularly for ILWBC living in rural or remote areas, as interventions are seldom implemented outside of academic or urban centers [6, 7].

Rural ILWBC are marginalized, facing distinctive challenges, including geographic isolation, limited healthcare infrastructure, lack of trained exercise specialists, transportation barriers and associated costs, and socioeconomic constraints [8, 9, 10, 11]. These factors reinforce longstanding inequities in supportive cancer care and may shape who enrolls in programs when access is offered. Yet little is known about how rural and urban ILWBC differ in demographic, medical, lifestyle, or functional profiles, or how these factors relate to enrollment. Without such data, it is difficult to design or scale interventions that equitably address the needs of diverse cancer populations [12, 13].

The Exercise for Cancer to Enhance Living Well (EXCEL) study offers a unique opportunity to examine and address this gap, delivering tailored exercise programs primarily to rural/remote ILWBC across Canada, with additional enrollment of urban participants who lacked exercise oncology resources during the COVID‐19 pandemic [14]. Leveraging this geographically diverse cohort, we conducted a geographically‐stratified secondary analysis examining baseline participant characteristics. Our purpose was to identify rural–urban differences that can inform diversity‐focused recruitment and guide sustainable implementation of exercise oncology programs.

## 2 Materials and Methods

### 2.1 Study Design

The EXCEL study is a five‐year hybrid effectiveness‐implementation trial that began in September 2020 and is ongoing through 2025. This pan‐Canadian initiative employs the RE‐AIM framework [15] to implement the delivery of an 8‐ to 12‐week structured exercise oncology program primarily online, with some in‐person offerings available in rural communities where feasible. EXCEL was designed to reach rural/remote populations (communities with < 100,000 people) as well as those living in urban populations where no existing exercise oncology resources are available. Rural–urban status was defined using a population‐based classification aligned with Statistics Canada Census Metropolitan Area criteria [16]. With the advent of the COVID‐19 pandemic, the EXCEL study expanded enrollment to urban participants who lacked access to exercise oncology resources [17], including individuals whose local programs were temporarily shut down during pandemic restrictions (March 2020–June 2021; Canadian Institute for Health Information, 2022 [18]). The inclusion of these “urban” populations (both underserved and during the COVID‐19 pandemic) was pragmatic, facilitating recruitment at cancer centers that included both urban and rural patients, and within the aim of the EXCEL study to provide exercise resources to underserved populations [14].

Participants were assessed at baseline, immediately following the intervention (8–12 weeks), and again at 24 weeks post‐intervention, with optional annual follow‐ups extending up to 5 years for patient‐reported outcomes. The present secondary observational analysis examines baseline characteristics data collected from participants enrolled between Fall 2020 and Spring 2025 in the EXCEL cohort study (Figure S1). This analysis focuses exclusively on enrolled participants at baseline to provide an understanding of potential similarities and differences in participant characteristics from rural and urban locations. These analyses will be used to guide the subsequent primary analysis of the effectiveness of the EXCEL trial. Ethics approval for the study was provided by the Cancer Control Health Research Ethics Board of Alberta (HREBA.CC‐20‐0098), with approvals also obtained at all participating regional hub sites across Canada.

### 2.2 Participants

**FIGURE 1:**
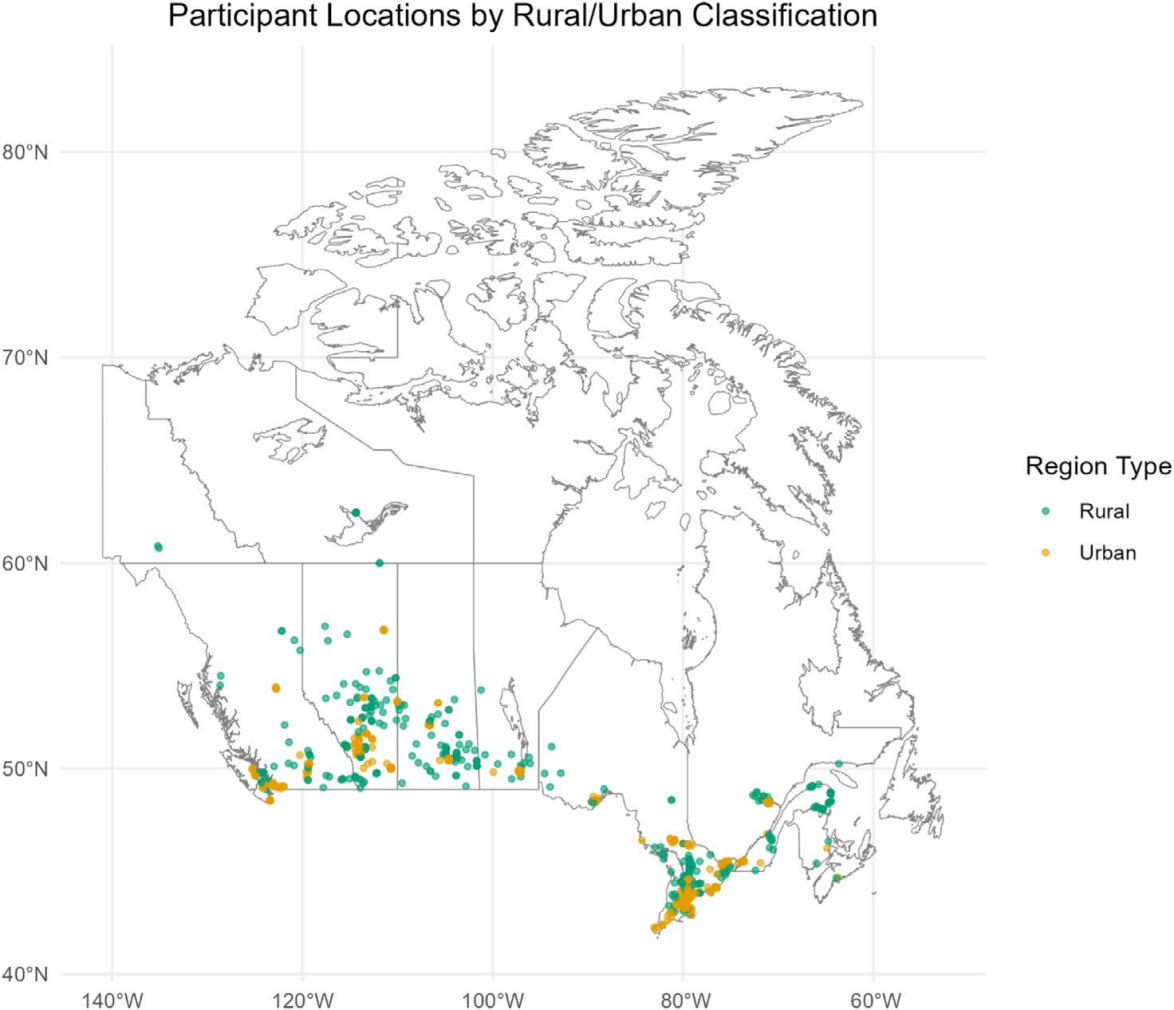
Anonymized rural vs. urban locations of participants in the EXCEL program, where postal codes were available.

Eligible participants for EXCEL were adults (≥ 18 years) with any cancer diagnosis who were pre‐treatment, actively receiving treatment, or up to 3 years post‐treatment (with allowances beyond 3 years via medical referral). Additional inclusion criteria included the ability to participate in mild physical activity twice weekly, residency in a rural area (population < 100,000), in an underserved urban center with no existing access to cancer exercise resources, or in an urban center where access was removed during the COVID‐19 pandemic period (2020–2021), reliable internet access for virtual class participation, and a working computer camera for safety monitoring during classes. Recruitment was coordinated through regional hub sites in Calgary, Halifax, Toronto, Vancouver, and Montréal, with participant screening performed by Clinical Exercise Physiologists (CEPs) [19]. Recruitment included self‐referral (from social media posts, posters or brochures circulated through clinical sites, or word of mouth) or via healthcare providers (HCPs). HCP referrals were classified as being directly referred from an HCP (the HCP obtained consent and sent the referral) or indirectly referred from an HCP (the HCP offered information, and the participant contacted the team of their own volition). Participants were enrolled from all provinces and several territories (see Figure 1). Screening involved a health history review and a Physical Activity Readiness Questionnaire (PAR‐Q+; [20]). Informed consent was obtained prior to study participation, and ethics approval was received from the Health Research Ethics Board of Alberta (HREBA.CC‐20‐0098).

### 2.3 Exercise Intervention

Details of the exercise intervention have been reported previously [14]. Briefly, participants attended twice‐weekly group exercise classes (8–15 individuals per class) over an 8–12‐week period, delivered by Qualified Exercise Professionals (QEPs). While the majority of sessions were delivered via Zoom, in‐person classes were also available in select rural locations within the Maritime provinces, Alberta, and Ontario [14]. In addition to physical training, the intervention incorporated an Exercise and Educate behavioral support model [14]. Education was delivered through printed handouts, brief discussions during class time, and optional biweekly live webinars, which were also made available as recordings.

### 2.4 Measures

Data used to characterize the participants at baseline included the following:

#### 2.4.1 Demographics and Lifestyle/Medical Characteristics

Comprehensive demographic and lifestyle data were collected to characterize individuals who were referred to and enrolled in the EXCEL program. These data provide important context for understanding who the program is reaching and act to inform interpretation of program accessibility and generalizability for ILWBC in rural and remote communities.

Upon enrolment, participants self‐reported key demographic information, including: age, sex, gender identity, ethnicity, education level, employment status, household income, and geographic location (postal codes were used to classify participants residences according to Statistics Canada's rural and remote designations, providing insight into geographic reach).

In addition to demographics, participants reported lifestyle and medical characteristics relevant to program engagement and health status. These included current physical activity levels modified‐GLTEQ; [21], cancer diagnosis and treatment history, and comorbidities.

#### 2.4.2 Cancer Impairments and Symptom Profile

Participants completed a suite of validated questionnaires electronically through the REDCap platform [22, 23] to assess demographics, lifestyle characteristics, quality of life, cognitive function, fatigue, symptom burden, and exercise‐related self‐efficacy including: ESAS‐r (Edmonton Symptom Assessment Scale—Revised): Rates 9 symptoms on a scale of 0–10, producing a total symptom burden score (range 0–90) [24].

#### 2.4.3 Health‐Related Fitness Measures

Physical assessments were primarily conducted virtually, supervised by two trained CEPs to ensure accuracy and participant safety. Measures included: BMI, calculated using self‐reported height and weight; shoulder flexion range of motion, measured in degrees (0–180) [25]; 30‐s sit‐to‐stand test, total repetitions completed in 30 s [26, 27]; sit‐and‐reach, assessed lower body flexibility in centimeters [28]; cardiovascular endurance, evaluated using either the 2‐min step test (online) [29] or the 6‐min walk test (in‐person); single‐leg balance, total time (in seconds) a participant could balance on one leg [30].

If physical limitations prevented completion of a test, the assessment was omitted. Specific details regarding each physical function assessment can be found in EXCEL's previously published protocol manuscript [14].

#### 2.4.4 Statistical Analysis

All analyses were conducted using RStudio. Descriptive statistics (means, standard deviations, frequencies, and percentages) were used to summarize the data. Given the non‐normal distribution of physical activity data, values were summarized using medians and interquartile ranges (IQR). To reduce the influence of extreme values, an IQR‐based approach was used to identify implausible observations (values below Q1–1.5 × IQR or above Q3 + 1.5 × IQR), calculated separately by rural–urban classification. Observations outside these bounds were excluded from summary analyses. As an exploratory assessment of baseline cohort composition, we conducted post hoc comparisons of selected participant characteristics by rural–urban status. Variables were chosen based on relevance to the study objective and included age, sex, educational status, and BMI. Differences in age were assessed using Welch's *t*‐tests due to unequal variances, sex and education using chi‐square tests, and BMI using Wilcoxon rank‐sum tests due to non‐normal distributions. These analyses were descriptive in nature and intended to contextualize observed rural–urban differences rather than to support causal inference or to test hypotheses.

## 3 Results

A total of 2743 individuals expressed initial interest in the study. Of those, 2741 were contacted for initial screening and 624 were either ineligible or did not proceed further (see Figure S2 for reasons). Of those that were successfully contacted, 2117 were eligible and willing to proceed after initial screening (see Figure S2 for details). Of those that completed pre‐screening, 18.5% were indicated as being directly referred from an HCP, 15.8% were indicated as being indirectly referred from an HCP, and 65.7% were indicated as being self‐referred to the study. The total number of participants enrolled in the EXCEL study was 1576, with 81 participants indefinitely delaying their intervention start after enrolling. Thus, the number of participants who participated in the EXCEL intervention was 1495.

### 3.1 Demographic Characteristics

Of the 1495 individuals enrolled in the EXCEL program, there were 1085 rural and 400 urban participants, representing diverse geographic regions across Canada (Table 1). The mean age of participants was similar between groups (overall: 59.7 ± 12.4 years; rural, 60.7 ± 12.3 years; urban, 57.2 ± 12.6 years). The majority of participants were female (79.4%), with less female participants in rural (77.8%) vs. urban (85.8%) groups.

**TABLE 1:** Demographic characteristics of EXCEL at baseline and Canada‐wide population estimates.

Demographic characteristic	Total cohort (*n = *1495)		Rural participants (*n* = 1085)^a^		Urban participants (*n* = 400)^a^	
	Mean	SD	Mean	SD	Mean	SD
Age years, mean, SD	59.7	12.4	60.7	12.3	57.2	12.6
Age groups	**Number**	**%**	**Number**	**%**	**Number**	**%**
17–39 years	111	7.4	65	6.0	46	11.5
40–49 years	229	15.3	151	13.9	78	19.5
50–59 years	341	22.8	243	22.4	98	24.5
60–69 years	434	29.0	330	30.4	104	26.0
70+ years	338	22.6	266	24.5	72	18.0
*Not reported*, *n*, %^b^	42	2.9	30	2.8	2	0.5
Female	1187	79.4	844	77.8	343	85.8
Male	268	17.9	213	19.6	55	13.8
*Not reported*, *n*, %^b^	40	2.7	28	2.6	2	0.4
Indigenous	57	3.8	40	3.7	17	4.3
African	7	0.5	7	0.6	0	0.0
Arab	6	0.4	1	0.1	5	1.3
British	593	39.7	471	43.4	122	30.5
Caribbean	11	0.7	3	0.3	8	2.0
East and South Asian	33	2.2	16	1.5	17	4.3
Eastern European	104	7.0	63	5.8	41	10.3
Latin/Central and South America	12	0.8	6	0.6	6	1.5
Northern European	43	2.9	33	3.0	10	2.5
Southern Asia	21	1.4	4	0.4	17	4.3
Southern Europe	26	1.7	14	1.3	12	3.0
Western Asia	2	0.1	0	0.0	2	0.5
Western European	223	14.9	168	15.5	55	13.8
Other or Multi‐ethnicity	218	14.6	161	14.8	57	14.3
*Not reported*, *n*, %^b^	139	9.3	98	9.0	31	7.8
Never married	115	7.7	78	7.2	37	9.3
Married or common‐law	1103	73.8	808	74.5	295	73.8
Divorced or separated	161	10.8	113	10.4	48	12
Widowed	76	5.1	58	5.3	18	4.5
*Not reported*, *n*, %^b^	40	2.7	28	2.6	2	0.5
Some high school	43	2.9	34	3.1	9	2.2
Completed high school	131	8.8	108	10.0	23	5.8
Some university/college	229	15.3	183	16.9	46	11.5
Completed university/college	710	47.5	505	46.5	205	51.2
Some graduate school	47	3.1	36	3.3	11	2.8
Completed graduate school	294	19.7	190	17.5	104	26.0
*Not reported*, *n*, %^b^	41	2.7	29	2.7	2	0.5
≤ CAD 20,000	56	3.7	39	3.6	17	4.2
CAD 20,000‐CAD 39,999	133	8.9	99	9.1	34	8.5
CAD 40,000‐CAD 59,999	213	14.2	168	15.5	45	11.2
CAD 80,000‐CAD 99,999	226	15.1	174	16.0	52	13.0
≥ CAD 100,0000	220	14.7	171	15.8	49	12.2
Missing/not reported	472	31.6	316	29.1	156	39.0
Not reported, *n*, %^b^	175	11.8	118	10.9	47	11.8

^a^ Rural and urban were classified as communities < 100,000 and > 100,000 respectively. *N* = 10 participants did not provide sufficient data for this classification.

^b^ Data not reported due to self‐reported entry by participants.

Participants reported a range of educational backgrounds, with 47.5% having completed university or college and 19.7% holding a graduate degree (Table 1). Graduate degree attainment was more common among urban participants (26.0%) compared to rural participants (17.5%). Household income data varied across the cohort, though a substantial proportion of respondents (31.6%) did not report income. See Table 1 for all demographic data.

### 3.2 Lifestyle and Medical Characteristics

The majority of participants (86.6%) reported a single primary cancer diagnosis, while 9.6% reported two primary cancers and 1.3% reported three or more. Breast cancer was the most common diagnosis (49.8%), with a higher prevalence among urban participants (60.8%) compared to rural participants (46.3%). Other common diagnoses included hematologic (8.9%), genitourinary (7.1%), lung (7.2%), and digestive cancers (6.3%). A substantial proportion of overall participants (22.3%) reported a form of advanced cancer or metastatic disease. Over half of the cohort (54.8%) was actively undergoing cancer treatment at the time of enrolment. See Table 2 for all medical characteristics.

**TABLE 2:** Cancer and treatment characteristics of EXCEL participants at baseline.

Medical characteristics	Total cohort (*n* = 1495)		Rural participants (*n* = 1085)		Urban participants (*n* = 400)	
	Number	*%*	Number	*%*	Number	*%*
Number of cancers diagnosed						
Single primary cancer	1295	86.6	931	85.8	363	90.1
Two primary cancers	143	9.6	115	10.6	27	6.8
Three+ primary cancers	20	1.3	14	1.3	6	1.5
^¥^ *Not reported*, *n*, %	37	2.5	25	2.3	4	1
Primary cancer type^a^						
Breast	745	49.8	502	46.3	243	60.8
Hematologic	133	8.9	109	10.0	24	6.0
Genitourinary	106	7.1	87	8.0	19	4.8
Digestive	94	6.3	81	7.5	13	3.3
Head and neck	15	1.0	13	1.2	2	0.5
Gynecologic	110	7.4	84	7.7	26	6.5
Neurological	25	1.7	19	1.8	6	1.5
Lung	107	7.2	69	6.4	38	9.5
Skin	53	3.5	38	3.5	15	3.8
Thyroid	7	0.5	5	0.5	2	0.5
Other	63	4.2	53	4.9	8	2.0
^¥^ *Not reported*, *n*, %	37	2.5	25	2.3	4	1.0
High risk status/confirmed metastatic disease^b^	333	22.3	267	24.6	78	19.5
^¥^ *Not reported*, *n*, %	41	2.7	31	2.9	0	0
Cancer treatment status						
On treatment	819	54.8	580	53.5	238	59.5
Off treatment	638	42.7	479	44.1	158	39.5
^¥^ *Not reported*, *n*, %	38	2.5	26	2.4	4	1.0
Cancer treatment completed‐ type						
Surgery	1033	33.9	735	33.7	298	34.3
Chemotherapy	802	26.3	588	27.0	213	24.5
Radiation therapy	766	25.1	536	24.6	230	26.4
Hormonal therapy	187	6.1	139	7.1	48	5.5
Targeted/biological	31	1.0	25	6.4	6	0.7
Other treatment	230	7.5	155	1.1	75	8.6

^a^ If one or more cancers were reported, the primary cancer was determined based on the study's eligibility criteria (either during treatment or within 3 years after completing treatment).

^b^ High risk includes cases such as multiple myeloma, cancers of the head and neck, lung, pancreas, primary brain tumors, or confirmed metastatic disease involving distant sites or organs.

At baseline, 55.7% of participants identified as never‐smokers, with a higher proportion of never‐smokers in urban participants (60.5%) compared with rural participants (54.4%). A higher proportion of participants in rural areas identified as ex‐smokers (40.1%) compared with urban participants (35.3%). Physical activity levels varied overall, with 59.0% of participants classified as physically active, 18.0% as insufficiently active, and 17.3% as physically inactive. These proportions were comparable across rural and urban groups. Participants reported a median of 385 min of physical activity per week [IQR 315], with more physical activity reported among rural participants (median 392 min; IQR 322) compared to urban participants (median 360 min; IQR 290). Overall, 78% of participants met or exceeded recommended physical activity guidelines [31], with similar proportions observed among rural (79%) and urban (76%) participants. See Table 3 for all lifestyle behavior data.

**TABLE 3:** Lifestyle behaviors, smoking, drinking, and physical activity for the EXCEL participants at baseline.

Lifestyle behavior	Total cohort (*n* = 1495)		Rural participants (*n* = 1085)		Urban participants (*n* = 400)	
	Number	*%*	Number	*%*	Number	*%*
Never smoker	832	55.7	590	54.4	242	60.5
Ex‐smoker	576	38.5	435	40.1	141	35.3
Occasional Smoker	31	2.1	20	1.8	11	2.8
Regular Smoker	16	1.1	12	1.1	4	1.0
^¥^ *Not reported*, *n*, %	40	2.7	28	2.6	2	0.5
Never drinker	129	9.2	79	7.3	50	12.5
Ex‐drinker	185	13.2	133	12.3	52	13.0
Social drinker	351	25.0	545	50.2	184	46.0
Regular drinker	60	4.3	245	22.6	106	26.5
^¥^ *Not reported*, *n*, %	41	2.9	29	2.7	2	0.5
Active	882	59.0	636	58.6	246	61.5
Insufficiently active	269	18.0	193	17.8	76	19.0
Physically inactive	258	17.3	194	17.9	64	16.0
^¥^ *Not reported*, *n*, %	86	5.8	62	5.7	14	3.5
Physical activity	**Mean**	**SD**	**Mean**	**SD**	**Mean**	**SD**
Minutes per week	470	293	479	286	447	312

### 3.3 Cancer Impairments and Symptom Profile

A wide range of cancer‐related impairments were reported on the ESAS‐r (see Table 4 for all variables). Cancer‐related fatigue was the most common, affecting 20.9% of participants, followed by muscle and joint issues (14.2%), peripheral neuropathy (10.8%), pain (10.0%), and cognitive challenges (10.0%). These impairments were similarly distributed across rural and urban groups. Symptom scores assessed using the ESAS‐r revealed moderate levels of tiredness (3.8 ± 2.5) and general well‐being (3.5 ± 2.5), with no notable differences between rural and urban participants.

**TABLE 4:** Self‐reported symptoms and impairments of EXCEL participants at baseline. Measures from ESAS‐r and self‐reported questionnaires.

Cancer impairments and symptom profile	Total cohort (*n* = 1495)		Rural participants (*n* = 1085)		Urban participants (*n* = 400)	
	Number	*%*	Number	*%*	Number	*%*
Bladder/bowel issues	281	5.7	215	6.1	66	4.7
Breathing issues	230	4.6	167	4.7	61	4.3
Cognitive challenges	497	10.0	357	10.1	140	9.9
Communication issues	84	1.7	58	1.6	26	1.8
Cancer‐related fatigue	1039	20.9	748	21.1	289	20.5
Cardiac issues	74	1.5	56	1.6	17	1.2
Lymphedema	255	5.1	166	4.7	89	6.3
Muscle/joint issues	704	14.2	492	13.9	211	15.0
Osteoporosis or bone loss	126	2.5	84	2.4	42	3.0
Ostomy	19	0.4	17	0.5	2	0.1
Pain	497	10.0	348	9.8	148	10.5
Peripheral neuropathy	536	10.8	391	11.0	145	10.3
Weight loss issues	453	9.1	324	9.1	129	9.2
Symptom: ESAS score	**Mean**	**SD**	**Mean**	**SD**	**Mean**	**SD**
Tiredness (lack of energy)	3.8	2.5	3.8	2.6	3.9	2.4
Pain	2.2	2.2	2.2	2.2	2.4	2.2
Drowsiness	2.7	2.5	2.7	2.6	2.6	2.5
Anxiety	2	2.3	1.9	2.3	2.3	2.3
Depression	1.8	2.3	1.8	2.3	1.9	2.2
Dyspnea	1.1	1.8	1.1	1.9	1.0	1.8
Lack of appetite	1.1	2.1	1.2	2.1	0.9	1.9
Nausea	0.5	1.3	0.5	1.3	0.4	1.1
General wellbeing	3.5	2.5	3.5	2.5	3.5	2.3

### 3.4 Health‐Related Fitness Measures

Baseline health‐related fitness measures indicated a broad range of functional capacities among participants (see Table 5 for all fitness measure data). BMI was similar between rural and urban participants (overall: 28.3 ± 6.2 kg/m^2^; rural: 28.6 ± 6.3 kg/m^2^; urban: 27.4 ± 6.0 kg/m^2^). Functional performance was comparable between groups, with rural participants completing an average of 12.5 ± 4.1 sit‐to‐stand repetitions and 68.9 ± 21.3 steps in the 2‐min step test, similar to urban participants (12.3 ± 3.8 and 66.4 ± 22.0, respectively). Balance and flexibility measures revealed small differences, with urban participants demonstrating marginally better performance in both domains.

**TABLE 5:** Health‐related fitness measures of EXCEL participants at baseline.

Health‐related fitness measures	Total cohort (*n* = 1495)		Rural participants (*n* = 1085)		Urban participants (*n* = 400)	
	Number	%	Number	%	Number	%
Body mass index category						
Underweight, *n*, %	25	1.7	16	1.5	9	2.3
Normal, *n*, %	401	26.8	269	24.8	132	33.0
Overweight, *n*, %	463	31.0	342	31.5	120	30.0
Obese class I (30–34.9 kg/m^2^), *n*, %	256	17.1	189	17.4	67	16.8
Obese class II (35–39.9 kg/m^2^), *n*, %	114	7.6	85	7.8	29	7.3
Obese class III or higher (≥ 40 kg/m^2^), *n*, %	66	4.4	56	5.2	10	2.5
^¥^ *Not reported*, *n*, %	170	11.4	128	11.8	33	8.3

	Mean	SD	Mean	SD	Mean	SD
	*n* = 1304		*n* = 940		*n* = 363	
Body mass index (kg/m^2^)	28.3	6.2	28.6	6.3	27.4	6.0
	*n* = 1284		*n* = 924		*n* = 359	
30 s sit‐to‐stand, no.	12.5	4.0	12.5	4.1	12.3	3.8
	*n* = 145		*n* = 138		*n* = 7	
6 MWT distance, *m* ^a^	473.9	191.9	480.7	193.3	340	93.8
	*n* = 1116		*n* = 766		*n* = 349	
2‐minute step test, number^a^	68.1	21.5	68.9	21.3	66.4	22.0
	*n* = 1192		*n* = 850		*n* = 342	
Sit and reach test, left, number	−1.8	13.1	−1.7	13.0	−2.2	13.3
Sit and reach test, right, number	−1.7	13.0	−1.6	12.9	−2.0	13.1
	*n* = 1304		*n* = 940		*n* = 363	
Shoulder flexion ROM right, degrees	158.6	20	158	21.3	160.3	15.8
Shoulder flexion ROM left, degrees	158.4	20.2	157.6	21.8	160.3	15.1
	*n* = 1293		*n* = 934		*n* = 359	
Single‐foot balance, right leg, seconds	29.1	16.8	28.3	17.0	31.2	16.2
Single‐foot balance, left leg, seconds	28.7	16.8	27.7	17.0	31.5	15.9

*Note:* BMI classifications determined according to Health Canada (Canadian Guidelines for Body Weight Classification in Adults. Ottawa: Minister of Public Works and Government Services Canada; 2003).

Abbreviations: 6MWT, six‐minute walk test; m, meters; ROM, range of motion.

^a^ Only those who completed assessments in person were able to complete the 6MWT. Online fitness assessments included the 2MST.

### 3.5 Exploratory Statistical Comparisons

Exploratory comparisons of key baseline characteristics by rural–urban status indicated significant differences in sex distribution, educational attainment, and BMI (all *p* < 0.01). Age did not differ significantly between rural and urban participants (*p* = 0.08).

## 4 Discussion

This article presents a geographically‐stratified baseline analysis of participants enrolled in the EXCEL exercise oncology program, which was designed to reach underserved ILWBC in both rural/remote and urban settings without access to exercise oncology resources. Exploratory comparisons of baseline characteristics identified several modest but statistically significant rural–urban differences, including sex distribution, educational attainment, and BMI, while age did not differ significantly between groups. Overall, rural participants were more often male and had lower educational attainment, whereas urban participants demonstrated greater ethnic diversity and higher levels of education. These patterns are consistent with broader Canadian rural–urban health and sociodemographic trends [32] and may inform considerations for geographic context when planning and implementing exercise oncology programs. Rural–urban disparities in cancer detection, stage at diagnosis, treatment access, and survival outcomes have been consistently reported, with rural residents facing structural barriers to early screening and specialty care [33]. This literature supports the notion that rural populations experience distinct cancer care contexts compared with urban patients, and these factors may influence the types of participants who use exercise oncology resources across both settings.

For example, some variation in medical characteristics by geography was observed. Urban participants were more frequently living with breast cancer, whereas rural participants were more often diagnosed with hematologic, digestive, and genitourinary malignancies and were slightly more likely to present with metastatic disease. Urban participants were also more often undergoing active treatment at enrollment. These patterns are consistent with previously reported rural–urban disparities in cancer detection and access to specialty care, including later‐stage diagnosis and differences in treatment pathways in rural populations [34]. In the context of exercise oncology, these findings suggest the importance of ensuring programs are built and delivered to address potential needs across a range of disease stages and treatment statuses (i.e., a tailored program approach that meets the needs of individuals within the group setting) [14], rather than suggesting fundamentally different programming by geography.

Lifestyle behaviors showed modest variation by geography. Urban participants were more often never smokers and never drinkers, although regular alcohol use was also more common. Notably, baseline physical activity levels were high in both rural and urban participants; approximately three‐quarters of participants met or exceeded recommended physical activity guidelines [31]. This level of baseline activity is higher than typically reported in population‐based cancer cohorts and exercise trials [35], suggesting possible selection of individuals already motivated or able to engage in physical activity. Prior research has also identified rural–urban differences in health behaviors including tobacco use and health outcomes such as obesity among ILWBC, with rural participants often reporting higher levels of inactivity and risk factors that may contribute to poorer long‐term outcomes [36, 37]. This potential participation bias should be considered when interpreting generalizability, particularly for populations facing greater structural or health‐related barriers to exercise engagement. Given the relatively low rates of healthcare provider referral, the implementation of systematic referral pathways may be especially important for building feasible and sustainable exercise oncology programs, as such approaches could engage individuals with lower physical activity levels or less experience who may not initially express interest in participation.

Symptom burden and cancer‐related impairments were broadly similar across rural and urban participants. The comparable prevalence of fatigue, pain, anxiety, and neuropathy suggests that assumptions regarding greater symptom burden in rural populations [38] may not be reflected within cohorts accessing exercise oncology programs. However, a recent study found rural and urban cancer survivors often report similar prevalence of symptoms and treatment side effects, with only modest differences, indicating that similar to our findings, symptom profiles may not differ markedly by geography in survivorship cohorts [39]. From an implementation perspective, this supports the feasibility of offering exercise oncology programming across rural–urban settings, and tailoring as needed based on participants’ characteristics and needs at baseline.

Rural participants had slightly higher BMI and a greater prevalence of overweight and obesity, consistent with established rural health patterns [33]. However, functional performance and fitness measures were largely comparable between groups. Taken together, these findings suggest that while rural participants may have a higher burden of cardiometabolic risk factors at the population level [33] they do not appear to enter exercise oncology programs with markedly lower functional capacity, perhaps as a function of the high overall physical activity levels. While rural–urban disparities exist in health and health behaviors, emerging work underscores the need for context‐sensitive but broadly accessible interventions that can be delivered across settings [33]. Rather than implying the need for distinct rural vs. urban programs, these observations highlight areas that may warrant further evaluation in future studies to support program engagement, adherence, and potential beneficial outcomes across these settings.

### 4.1 Strengths

This study leverages baseline data from a large national exercise oncology trial, providing insight into demographic and physical activity differences between rural and urban ILWBC. The inclusion of participants from rural and remote settings addresses a longstanding gap in exercise oncology research, which has largely focused on urban populations [40]. Understanding baseline characteristics of the study participants supports the continued integration of EXCEL in a clinic‐to‐community model that reduces access gaps to exercise oncology resources for ILWBC. Reporting baseline demographic, lifestyle, medical, health‐related fitness, and symptom characteristics highlights geographic variation that may be used to support future resource development, from educational resources to both online and in‐person community‐based exercise oncology programs. Investigating the impact of potential baseline differences on program outcomes (i.e., effectiveness) will be explored in the complete EXCEL dataset and may inform the design of future community‐based exercise oncology programs.

### 4.2 Limitations

A limitation of the current analysis is that rural–urban location was not obtained with expressions of interest or initial eligibility screening. While such data may support designing recruitment strategies specific to geographic location in future scaled implementations of exercise oncology programming, the current findings do provide an understanding of demographic‐based differences in the rural–urban participants that can aid in tailoring future outreach. A further limitation is the reliance on self‐report measures for our lifestyle and medical characteristics data that introduces potential reporting bias, as there was no ability to complete a health records check to verify the data [41]. However, comprehensive intake procedures by the hub teams CEPs ensured participants completion and verification of the information provided. In addition, baseline fitness assessments were incomplete for a portion of participants, which may have limited our interpretation of functional capacity across groups. Regarding our geographical stratification, we acknowledge that our population‐based rural–urban classifications encompass heterogeneous settings and may not fully capture gradients of remoteness. However, this approach aligns with Canadian standards [16] and is appropriate for examining program reach and implementation across large versus smaller population centers. Additionally, although some rural–urban differences were statistically detectable, most were modest in magnitude, suggesting potential limited clinical relevance at the individual level. Despite these limitations, this analysis provides valuable insight into the characteristics of participants in the EXCEL study.

## 5 Conclusion

In summary, this study describes rural–urban variations in sociodemographic, medical, and lifestyle characteristics among individuals beginning an exercise oncology program, while symptom burden, physical activity levels, and functional capacity were largely comparable. Despite modest differences in age, education, BMI, and cancer characteristics, participants across geographies demonstrated similarly high physical activity levels and thus readiness for exercise, highlighting the need for continued evaluation of inclusive recruitment strategies and program reach to ensure broad population representativeness.

## Author Contributions

**Jonathan L. Low:** conceptualization (lead), data curation (equal), formal analysis (lead), investigation (lead), methodology (lead), project administration (equal), resources (equal), validation (equal), visualization (equal), writing – original draft (lead), writing – review and editing (lead). **Julianna Dreger:** data curation (equal), funding acquisition (equal), investigation (equal), methodology (equal), project administration (equal), resources (equal), validation (equal), writing – review and editing (equal). **Chad W. Wagoner:** data curation (equal), formal analysis (supporting), investigation (equal), project administration (equal), resources (equal), validation (equal), writing – review and editing (equal). **Emma McLaughlin:** data curation (equal), investigation (equal), validation (equal), writing – review and editing (equal). **Margaret L. McNeely:** conceptualization (equal), funding acquisition (equal), investigation (equal), project administration (equal), resources (equal), supervision (equal), writing – review and editing (equal). **Melanie R. Keats:** conceptualization (equal), funding acquisition (equal), investigation (equal), project administration (equal), resources (equal), supervision (equal), validation (equal), writing – review and editing (equal). **Daniel Santa Mina:** conceptualization (equal), funding acquisition (equal), investigation (equal), project administration (equal), software (equal), supervision (equal), validation (equal), writing – review and editing (equal). **Linda Trinh:** conceptualization (equal), funding acquisition (equal), investigation (equal), project administration (equal), resources (equal), supervision (equal), validation (equal), writing – review and editing (equal). **Kristin L. Campbell:** conceptualization (equal), funding acquisition (equal), investigation (equal), project administration (equal), resources (equal), supervision (equal), validation (equal), writing – review and editing (equal). **Isabelle Doré:** conceptualization (equal), funding acquisition (equal), investigation (equal), project administration (equal), resources (equal), supervision (equal), validation (equal), writing – review and editing (equal). **Heejae Lee:** conceptualization (equal), formal analysis (equal), investigation (equal), validation (equal), visualization (equal), writing – original draft (equal), writing – review and editing (equal). **Colleen A. Cuthbert:** funding acquisition (supporting), supervision (supporting), writing – review and editing (supporting). **Lauren C. Capozzi:** funding acquisition (supporting), resources (supporting), writing – review and editing (supporting). **Daniel Sibley:** data curation (equal), investigation (equal), validation (equal), writing – review and editing (equal). **Thomas B. Christensen:** data curation (equal), investigation (equal), methodology (equal), resources (equal), validation (equal), writing – review and editing (equal). **Alexia Piché:** data curation (equal), investigation (equal), methodology (equal), resources (equal), validation (equal), writing – review and editing (equal). **Kelly Mackenzie:** data curation (equal), funding acquisition (equal), project administration (equal), resources (equal), supervision (equal), writing – review and editing (equal). **Carol Yin:** data curation (equal), formal analysis (equal), methodology (equal), resources (equal), validation (equal), writing – review and editing (equal). **S. Nicole Culos‐Reed:** conceptualization (lead), data curation (equal), formal analysis (equal), funding acquisition (lead), investigation (lead), methodology (lead), project administration (lead), resources (lead), supervision (lead), validation (equal), visualization (equal), writing – original draft (lead), writing – review and editing (lead).

## Funding

This work was supported by a Canadian Institutes of Health Research and Canadian Cancer Society Survivorship Team Gran (Grant # 706673). Additional program funding provided by the Alberta Cancer Foundation (Grant #N/A). SNCR is a UCalgary Research Excellence Chair and a Killam Laureate. JLL holds an Alberta Innovates Post‐Doctoral Fellowship and a Riddell Centre for Cancer Immunotherapy Post‐Doctoral Award, Arnie Charbonneau Cancer Institute.

## Conflicts of Interest

The authors declare no conflicts of interest.

## Supporting information


**Supplementary Figure 1** The EXCEL study timeline.

**Supplementary Figure 2** Consort diagram for the EXCEL study.

## Data Availability

Data can be made available upon reasonable request.
