# Supplementary figures and images for: Baseline Characteristics of Participants in the Exercise for Cancer to Enhance Living Well (EXCEL) Study: A Canada‐Wide Rural–Urban Analysis
Source: Cancer Med. 2026 Mar 20;15(3):e71629. doi: 10.1002/cam4.71629 (PMC13093277; doi:10.1002/cam4.71629)

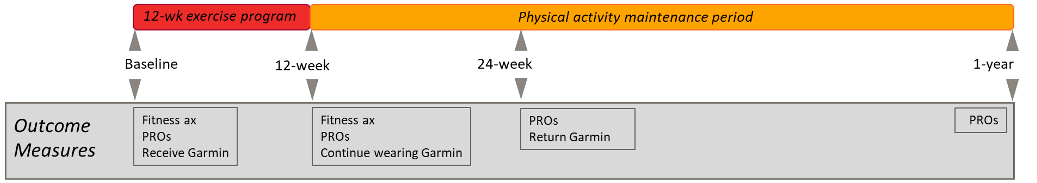

Supplement: Supplementary file 1 — Supplementary Figure 1 The EXCEL study timeline. [file CAM4-15-e71629-s002.png]

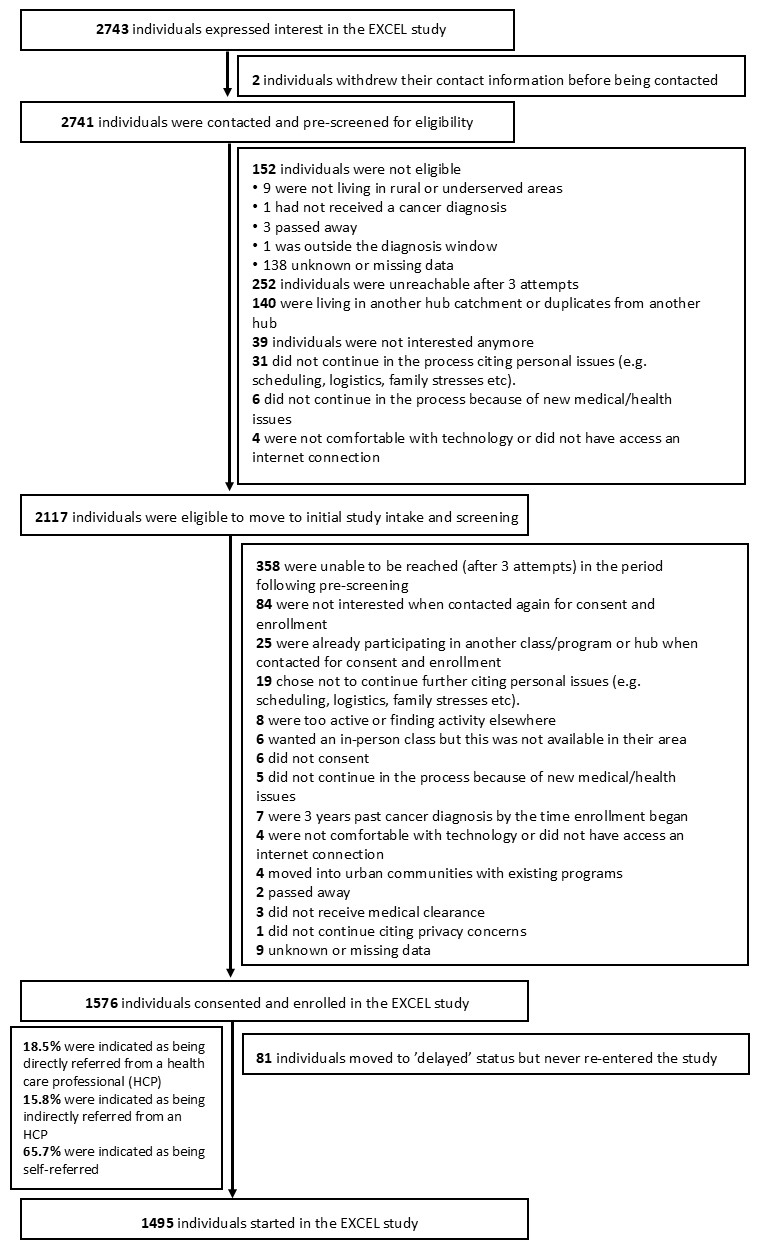

Supplement: Supplementary file 2 — Supplementary Figure 2 Consort diagram for the EXCEL study. [file CAM4-15-e71629-s001.jpg]
